# Incorporating public values into public health communications: Effects of value affirmations on intentions to vaccinate and trust in the U.S.

**DOI:** 10.1371/journal.pone.0356783

**Published:** 2026-09-16

**Authors:** Christopher Wolsko, Elizabeth Marino

**Affiliations:** 1 Department of Psychology, Oregon State University – Cascades, Bend, Oregon, United States of America; 2 Department of Anthropology, Oregon State University – Cascades, Bend, Oregon, United States of America; University of Stavanger, NORWAY

## Abstract

A set of COVID-19 pro-vaccine messages that aligned with key social values of vaccine-hesitant individuals was developed. In Spring of 2021, unvaccinated adults in the U.S. (*n* = 1,170) completed an online survey in which they were randomly assigned to read through one of several different pro-vaccine messages, followed by assessments of their future COVID-19 vaccine likelihood and level of trust in government, health care, and vaccines. Relative to a control condition, messages that affirmed individuals’ distrust in the government and concerns over restrictions on personal liberty resulted in *higher* vaccination likelihood and *greater* trust. While the observed effect sizes of these messaging conditions were small, the findings have implications for broadening the value frames of public health appeals and understanding how and why vaccine hesitancy acts as a powerful way for people to express their values and authentic experiences.

## Introduction

Effective management of public responses to the COVID-19 pandemic was challenged by numerous factors, including the inherent uncertainty and severity of the evolving crisis, communication inefficiencies and inconsistencies [1,2], propagation of mis- and disinformation [3], and low levels of trust between diverse members of the public, the biomedical and pharmaceutical industries, public health institutions, and government entities [4]. Determining the most efficient and persuasive way to convey essential public health information is a perennial puzzle, and vaccine communication is no exception, as the pandemic brought into sharp relief.

Past models that have been utilized to predict vaccination and other health behaviors, such as the Health Belief Model [5] and the Theory of Planned Behavior [6], have been criticized as overly individualistic and cognitively oriented, emphasizing perceived risks and benefits and narrow conceptualizations of social influence, while underestimating the complex roles of social identity, political polarization, and institutional trust in shaping behavior [7,8]. Conventional models have also failed to account for motivated reasoning and affective processes that can frequently override the influence of factual information [9–11]. Indeed, we no longer assume that people are essentially rational decision-makers when it comes to health care, or other domains [12–14], and research testing vaccination messaging techniques confirms this, demonstrating that simple fact-based communications are frequently ineffective [15,16].

Collectively, the trajectory of this literature suggests that more integrative approaches which incorporate important social and affective processes are needed to better explain and predict vaccine behavior. Cognizant of this, we set out during the initial public rollout of the COVID-19 vaccines in the U.S. in early 2021 to better understand some of the social factors that would predict whether or not people decided to get vaccinated. We were particularly interested in investigating how the affirmation of particular social identities and values would contribute to pro-vaccine intentions, and potentially increase institutional trust – an element that takes center stage in the confidence aspect of Betsch’s influential 5/7C model [17] and has been found to be predictive in many studies of vaccination behavior [7,18,19].

While research into public health messaging strategies has been dominated by a focus on gain vs. loss framing [20], a more recent, and promising, area of investigation has examined the effects of salient social identities and/or values present in messages [21–23]. In their review of randomized controlled vaccine communication trials, Xia and Nan [24] found that the most persuasive messages tended to be those that highlighted a shared identity between the message source and the audience, and a few studies have found that framing messages in terms of specific values that are similar to those of the audience may improve vaccine uptake [25,26]. This general strategy of pairing the social values in the message with the social values of the audience has also shown promise by reducing polarization and resistance to other contentious issues in the public square, including removing firearms as a suicide prevention strategy in the U.S. [27,28] and taking conservation action in the face of climate change skepticism [29,30].

The mechanisms behind public health attitude shifts of this type may be usefully interpreted in light of persuasion research on matching effects. Research in this area indicates that attitude and behavior change will be greater when persuasive communications are congruent with the content, structure, and/or function of the recipient's attitude in the relevant domain [31]. That is, the use of specific identity or value frames to alter attitudes appears to be effective, at least in part, by virtue of its success in matching the self- or value-expressive functions of respondents’ attitudes.

In highly politicized and contentious domains, such as with COVID-19 policy and vaccine attitudes in the U.S., individuals’ expressions of their attitudes are about far more than a particular attitude object – a vaccine in this case. Instead, attitudes serve important self-expressive functions, acting as vehicles for conveying social identity, lived experiences, and important social or moral values [9]. From this perspective, a person’s expression of an attitude against getting a COVID-19 vaccine may be seen as an affirmation of a particular social experience or value – for example, distrust of the government or of the health care system.

Just as an attitude may persist because it meets a functional need, so to, “attitude change occurs to meet a functional need” (31, p. 194). In the present context, we expected that the attitudes of the unvaccinated may change in the pro-vaccination direction under conditions in which specific self- or value-expressive functions are actually being fulfilled by agreeing with a pro-vaccine agenda (rather than being stifled, threatened, or deemed irrational by agreeing with an anti-vaccine agenda).

To investigate this general hypothesis, we developed a set of COVID-19 pro-vaccine messages that aligned with key social values that appeared to be critical for peoples’ decision to vaccinate, especially for those who were hesitant. We then conducted two separate experimental tests of the effects of these messages on the likelihood of non-vaccinated individuals getting a COVID-19 vaccine in the future, and on their level of distrust in government, health care, and vaccines.

## Materials and methods

### Participants and procedural overview

Two separate surveys were conducted during the Spring of 2021, just as the first major waves of people in the U.S. were getting vaccinated. All participants were adult U.S. residents who had pre-enrolled in the Amazon MTurk system. MTurk is an online labor market that has been widely utilized by survey researchers in psychology and other social sciences, and samples obtained are generally demographically diverse and display strong psychometric properties [32,33]. Individuals enrolled in the MTurk system were invited to complete one of two short, 10–15 minute, anonymous surveys in exchange for $1.00. All individuals were instructed to complete the survey only if they had *not* received a vaccination for COVID and they had *not* made an appointment to do so.

Survey 1 was conducted on April 1, 2021, and had 371 participants who were randomly assigned to one of four messaging conditions. Survey 2 was completed on May 24, 2021, and had 799 participants who were randomly assigned to one of five messaging conditions. Based on prior research indicating very small to small effect sizes for the influence of persuasive messaging on vaccine intentions and other health attitudes [34–36], our power analyses indicated the need for a minimum of 65 participants per condition. Because there was such well-publicized resistance to the rollout of COVID vaccines at the time, we expected that attitudes among the unvaccinated would be fairly resistant to change, and so raised our *n* per condition by 50%, to around 90 participants in Survey 1. For Survey 2 (conducted almost two months later), we anticipated that potential messaging effect sizes would be even weaker among the smaller population of unvaccinated adults, and so we almost doubled our sample size per condition to approximately 160.

Participants in both surveys were first randomly assigned to read information either from a control condition or from one of several vaccine messaging conditions. Following, individuals in both surveys responded to the following assessments in this order: COVID vaccine attitudes; distrust in vaccines, government, and the health care system; and demographics. Condition and key variable names are italicized in the descriptions that follow.

### Ethics statement

These surveys were administered as part of a program evaluation of COVID-19-related public health communications at the Deschutes County Health Department in 2021. The data was archived after this program evaluation and then later approved for analysis as research in 2022 by the Human Research Protection Program and Institutional Review Board at Oregon State University (IRB-2022–1357).

### Vaccine messaging conditions

To determine the content of the social values for the vaccine messages, we conducted a series of focus group interviews with vaccine-hesitant adults in the U.S. during the late winter and early spring of 2021. We sought to understand their most pressing concerns and to probe the perceptions of risk, cultural identities, and moral values that underpinned their COVID-19-related attitudes and behaviors. The dominant themes in our discourse analysis were distrust in sociopolitical and medical institutions, concerns about one’s ability to exercise personal liberty and freedom of choice (including whether or not to get a vaccine), and desires to protect and reconnect with family. From this analysis, we developed four distinct messaging slogans: (1) *government* – “You don’t have to trust the government, here are the facts,” which affirmed individuals’ social experience of feeling like politicians and governmental organizations are not trustworthy; (2) *families* – “Everybody wants to get back to their families,” which affirmed the importance of family as an identity and of people’s concerns about maintaining normal family life; and (3) *choice* – “It’s your life, it’s your choice” and (4) *liberty* – “COVID and politicians: Don’t tread on me,” which affirmed the values of personal liberty and freedom.

In Survey 1, participants were randomly assigned to read through one of 4 different messages: *control*; *families*; *government*; and *choice* (see S1 Appendix for the full text of each message). In the *control* condition, participants read the following statement: “First, consider the following information about the COVID-19 vaccines from physicians at the Mayo Clinic and the Centers for Disease Control and Prevention (CDC).” They were then presented with one page of actual information from these organizations that detailed the safety, efficacy, and rationale for the COVID-19 vaccines (see S1 Appendix). In the other 3 conditions, just prior to reading this same information about the COVID-19 vaccines from the Mayo Clinic and the CDC, participants were randomly exposed to either the *families*, *government*, or *choice* slogan, as described above, in large, bold font. Participants in Survey 2 were randomly assigned to one of the same four conditions used in Survey 1, *or* to the *liberty* slogan.

### COVID vaccine attitudes

Participants in both surveys responded to two items that assessed the likelihood of getting a COVID vaccine in the future. *Vaccine likelihood* was assessed with the question, “How likely are you to get an FDA authorized vaccine for COVID-19, once it is available to you for free?” Participants responded to this item on a 6-point scale, ranging from 1, *I will definitely NOT get the vaccine,* to 6, *I am certain that I’ll get the vaccine*. Additionally, in Survey 2, *child vaccine likelihood* was measured for parents of children under the age of 18 with the question, “How likely is it that you will have your child get an FDA authorized vaccine for COVID-19, once it is available for free? If you have more than one child under 18, answer for your youngest.” Parents responded to this item on a 6-point scale, ranging from 1, *I will definitely NOT have my child get the vaccine*, to 6, *I am certain that I’ll have my child get the vaccine.* This type of vaccine likelihood scale has been well-validated in a large number of studies assessing vaccine attitudes and behavioral intentions [37–39].

From a psychometric standpoint, we favored the continuous measurement of vaccine attitudes with our 6-point *likelihood* scale, but we also thought it was important from a policy perspective to examine our messaging effects with a second single-item assessment of vaccine intention that was utilized by the Kaiser Family Foundation (KFF) – who conducted the most frequent nationally representative tracking surveys of U.S. public opinion on COVID-19 issues at that time, and were a key barometer for media, policymakers and researchers at the time [40]. For this item, *vaccine intention* was assessed by asking participants the question, “When an FDA authorized vaccine for COVID-19 is available to you for free, do you think you will…?” and instructing them to select one of four response options: get vaccinated as soon as possible; wait and see; get vaccinated only if it’s required for my work or travel; or definitely not get vaccinated. We replicated the original KFF item and response options verbatim to preserve measurement comparability with this benchmark survey and to estimate the representativeness of our samples in terms of vaccine intentions.

### Distrust

Next, participants responded to three items designed to assess their level of distrust in vaccines (“I don't trust vaccines in general”), the government (“I don’t trust the government to make sure the COVID vaccines are safe and effective”), and the health care system (“I don't trust the health care system”). Responses were provided on a 7-point scale, ranging from 1, *strongly disagree*, to 7, *strongly agree*. The items were strongly intercorrelated, and a principal components factor analysis suggested a clear one-factor solution, so we decided to combine them into a single index of *health-related distrust* (α = .87 for Survey 1; α = .83 for Survey 2), by taking the mean for each participant across all three items, with higher numbers indicating reflecting *less* trust. This measure was modeled on items from prior research on trust in vaccines and aspects of the health care system [7,41]. The items have considerable face validity, and these elements of distrust have been shown to be highly predictive of vaccine attitudes [7,19].

### Demographics

Finally, participants responded to a series of demographic questions, in which they were asked to indicate their gender, age, highest level of formal education, annual household income, race/ethnicity, and political orientation. Political orientation was assessed by asking participants to indicate the degree to which they identified as more liberal or more conservative. Responses were obtained on a 7-point scale (1 = *extremely liberal*; 2 = *moderately liberal*, 3 = *slightly liberal*; 4 = *moderate*; 5 = *slightly conservative*; 6 = *moderately conservative*; 7 = *extremely conservative*), and each individual received a *political orientation* score based on their response to this item.

### Statistical analysis

All statistical analyses were conducted on SPSS Version 29. Missing responses for individual variables were minimal (<0.3% per survey) and were uncorrelated with demographics. Participants with missing data were excluded listwise from analyses. All tests were two-tailed and considered significant at *p* < .05. Effect sizes are reported in relevant tables.

Pearson correlation coefficients were computed to examine zero-order relationships between continuous variables. One-Way ANOVAs with Tukey post-hoc tests were conducted to examine any relationships between a continuous variable and a categorical variable with more than two-levels.

### Analysis of messaging condition effects

To make the relevant tests of condition effects on key dependent variables, we constructed one set of dummy codes for each survey, with the control condition as the reference category. In Survey 1, the 3 codes compared vaccine attitudes for: (1) *control vs. families;* (2) *control* vs. *government;* and (3) *control* vs. *choice*. In Survey 2, the same 3 comparisons were made, along with an additional code for *control* vs. *liberty*.

For each key dependent variable, we conducted two separate regression analyses: one in which the dependent variable (*vaccine likelihood*, *child vaccine likelihood, vaccine intention, or health-related distrust*) was regressed on to just the set of dummy codes (Model 1), and one in which the vaccine attitude was regressed on to the set of dummy codes along with three significant demographic predictors (political orientation, income, and education), as specified in the Results section, below (Model 2). We included this second model with covariates to increase the power of detecting condition effects [42,43], especially given the prominent role of political orientation in this domain of health behavior.

Linear regressions were conducted to predict responses on the continuous variables of *vaccine likelihood*, *child vaccine likelihood, and health-related distrust.* Multinomial logistic regressions were conducted on *vaccine intention*, as is standard for predicting differences in variables with more than two unordered categories.

## Results

### Participant demographics and representativeness

The sample for Survey 1 was relatively balanced in gender (48.8% male, 51.2% female); majority Caucasian (66.8% White, 15.1% Black/African American, 5.1% Latino/Hispanic, 10.5% Asian American, 0.8% Pacific Islander; 0.8% American Indian / Alaska Native); diverse in age (*M* = 38.29, *SD* = 11.58; range: 18–76 years old); and diverse in highest educational attainment (0.5%, some high school; 6.5%, high school diploma or GED; 23.2%, some college or associates degree; 45.3%, bachelor’s degree; 24.5%, master’s degree or higher) and household income (9.7%, less than $25,000; 24.5% from $25,000 to $49,999; 22.9% from $50,000 to $74,999; 23.2% from $75,000 to $99,999; 19.7%, $100,000 or more).

Demographic characteristics for Survey 2 were similar to those for Survey 1, except for a slightly greater percentage of male respondents (58.5% male, 41.5% female). The majority were Caucasian (66.7% White, 20.2% Black/African American, 4.9% Latino/Hispanic, 5.6% Asian American, 0.4% Pacific Islander; 1.6% American Indian / Alaska Native); and the sample was diverse in age (*M* = 36.95, *SD* = 10.86; range: 18–80 years old), highest educational attainment (0.4%, some high school; 6.9%, high school diploma or GED; 20.8%, some college or associates degree; 49.9%, bachelor’s degree; 22.1%, master’s degree or higher) and household income (9.9%, less than $25,000; 24.9% from $25,000 to $49,999; 31.2% from $50,000 to $74,999; 19.3% from $75,000 to $99,999; 14.6%, $100,000 or more).

Taken together, and relative to U.S. census data from the time of the study [44], our samples were fairly representative of gender, slightly underrepresented the Latino/Hispanic population, were slightly younger relative to the average U.S. adult, and were somewhat more educated and less wealthy.

To estimate the representativeness of our sample in terms of vaccine attitudes, we compared responses on our *vaccine intention* variable from participants in our control conditions (see Table 1) to those collected from nationally representative samples during the same months with the same exact survey item by the Kaiser Family Foundation [40]. For Survey 1, the relative percentages of participants’ responses to the categories of the *vaccine intention* variable were all within a few percentage points of the nationally representative data. For Survey 2, the percentage of our respondents who said they would get a vaccine as soon as possible (40.7%) was substantially higher than in the nationally representative data at the time (20.9%), and the percentage of respondents who said they would definitely not get it (11.7%) was substantially lower than in the nationally representative data (30.2%). Thus, our Survey 2 sample was less vaccine hesitant than the general population at the time.

**Table 1:** Means and frequencies for vaccination attitudes by condition and survey.

Survey 1								
*Condition*	*Vaccine Likelihood*		*Vaccine Intention*					
		Get it ASAP	Wait and See	Only if Required	Definitely Not		*Distrust*	
Control (*n*=93)	3.82	49.5%	21.5%	10.8%	18.3%		3.91	
Government (*n*=93)	4.33	65.6%	19.8%	7.3%	7.3%		3.65	
Families (*n*=96)	4.33	58.1%	24.7%	11.8%	5.4%		3.51	
Choice (*n*=89)	4.15	55.1%	25.8%	7.9%	11.2%		3.92	
**Survey 2**								
***Condition***	***Vaccine Likelihood***	***Child Vaccine Likelihood***	***Vaccine Intention***					
			**Get it ASAP**	**Wait and See**	**Only if Required**	**Definitely Not**		***Distrust***
Control (*n*=162)	3.31	3.04	40.7%	29.6%	17.9%	11.7%		4.37
Government (*n*=155)	3.78	3.32	45.2%	27.1%	18.7%	9.0%		4.26
Families (*n*=170)	3.46	3.23	39.4%	30.0%	15.9%	14.7%		4.23
Choice (*n*=156)	3.51	3.25	39.0%	24.0%	20.5%	16.4%		4.30
Liberty (*n*=163)	3.71	3.55	48.5%	34.4%	8.0%	9.2%		3.84

*Notes*: Means are presented for *vaccine likelihood*, *child vaccine likelihood,* and *distrust.* Likelihood variables were assessed on a 6-point scale, with higher numbers indicating greater likelihood. *Distrust* was assessed on a 7-point scale, with higher numbers indicating more health-related distrust. Percentages represent relative frequencies within condition for *vaccine intention*.

### Demographic correlates of vaccine attitudes

We first examined the extent to which, across all conditions of the surveys, our primary dependent variables of *vaccine likelihood* and *vaccine intention* were associated with participants’ demographic characteristics. Results indicated that political orientation, education, and income were the only demographics to emerge systematically as reliable predictors of vaccine attitudes. In the service of brevity, we highlight just a few of the findings. Participants who expressed a greater likelihood of obtaining a COVID-19 vaccine (higher scores on *vaccine likelihood*) also reported a higher income (Survey 1, *r*(369)=.16, *p* = .001; Survey 2, *r*(792)=.11, *p* = .002), a higher level of education (Survey 1, *r*(369)=.10, *p* = .049; Survey 2, *r*(792)=.13, *p* < .001), and a greater tendency toward politically liberalism (Survey 1, *r*(368)=−.31, *p* < .001; Survey 2, *r*(790)=−.23, *p* < .001).

### Experimental effects of messages on vaccine attitudes

The means (and frequencies, where relevant) for the vaccine attitude variables as a function of experimental condition and survey are presented in Table 1. Our predictions were that relative to the control condition, messages that affirmed individuals’ social experiences and values would precipitate a greater probability of vaccination.

#### Vaccine likelihood.

Findings from the regression analyses for *vaccine likelihood* are presented in Table 2. In Survey 1, the *control vs. families* coefficient and the *control vs. government* coefficient were both significant in Model 1 and in Model 2. This indicated that participants reported being more likely to get a COVID vaccine in the *government* condition and in the *family* condition than in the *control*. The *control vs. choice* coefficient was not significant in either Model.

**Table 2:** Regression analyses predicting vaccine likelihood with messaging conditions and demographic covariates.

	Model 1				Model 2			
Variable	*β*	*SE*	*t*	*p*	*β*	*SE*	*t*	*p*
Survey 1								
*Condition Codes*								
Control vs. Families	.13	.26	1.98	.048	.17	.25	2.82	.005
Control vs. Government	.13	.26	2.00	.046	.13	.24	2.13	.034
Control vs. Choice	.08	.26	1.25	.212	.09	.25	1.45	.149
*Demographic Covariates*								
Political Orientation					-.34	.04	-6.86	<.001
Income					.14	.06	2.61	.009
Education					.07	.10	1.25	.211
Survey 2								
*Condition Codes*								
Control vs. Families	.04	.18	0.82	.411	.03	.18	0.79	.429
Control vs. Government	.11	.19	2.52	.012	.10	.18	2.37	.018
Control vs. Choice	.05	.19	1.08	.281	.04	.18	0.98	.326
Control vs. Liberty	.10	.19	2.18	.029	.09	.18	1.97	.049
*Demographic Covariates*								
Political Orientation					-.24	.03	-7.09	<.001
Income					.09	.04	2.39	.017
Education					.14	.07	4.04	<.001

In Survey 2, the *control vs. government* coefficient and the *control vs. liberty* coefficient were significant in both Model 1 and in Model 2. This indicated that participants reported being more likely to get a COVID vaccine in the *government* condition and in the *liberty* condition than in the *control*. Neither the *control vs. families* contrast, nor the *control vs. choice* contrast, were significant in either Model.

#### Child vaccine likelihood.

Results from the regression analysis of *child vaccine likelihood* from Survey 2 are presented in Table 3. Only the *control vs. liberty* coefficient was a significant predictor of *child vaccine likelihood*. In both Models, parents reported being more likely to have their child get a COVID vaccine in the *liberty* condition than in the *control*. None of the other condition contrasts were significant.

**Table 3:** Regression analyses predicting child vaccine intentions with messaging conditions and demographic covariates (Survey 2).

	Model 1				Model 2			
Variable	*β*	*SE*	*t*	*p*	*β*	*SE*	*t*	*p*
*Condition Codes*								
Control vs. Families	.05	.19	1.01	.314	.05	.19	0.99	.324
Control vs. Government	.07	.20	1.44	.152	.06	.19	1.32	.189
Control vs. Choice	.05	.20	1.04	.298	.04	.20	0.93	.353
Control vs. Liberty	.12	.20	2.59	.010	.11	.19	2.40	.017
*Demographic Covariates*								
Political Orientation					−.18	.03	−7.09	<.001
Income					.07	.05	2.39	.075
Education					.15	.07	4.04	<.001

### Vaccine intention

Multinomial logistic regressions were conducted in order to analyze responses on the *vaccine intention* variable. Findings are presented in Table 4. Participants who indicated they would get the vaccine “as soon as possible” constituted the reference category for all analyses. In Survey 1, the *control vs. families* and the *control vs. government* contrasts significantly predicted a difference in relative frequencies of respondents who said they would get the vaccine “as soon as possible” vs. those who said they would “definitely not” get the vaccine. Looking back at the raw frequencies in Table 1, we can see that relative to the *control* condition, individuals in the *families* and the *government* conditions exhibited an increase in “as soon as possible” responses and a decrease in “definitely not” responses.

**Table 4:** Multinomial regression analyses predicting vaccine intention with messaging conditions and demographic covariates.

	Model 1					Model 2				
Variable	*B*	*SE*	*Wald*	*p*	*Exp(B)*	*B*	*SE*	*Wald*	*p*	*Exp(B)*
Survey 1										
*Wait and See*										
Control vs. Families	-.02	.37	.00	.955	.98	-.27	.39	.48	.488	.76
Control vs. Government	-.37	.37	.95	.329	.69	-.38	.39	.95	.330	.68
Control vs. Choice	.08	.37	.04	.835	1.08	.07	.39	.04	.851	1.08
Political Orientation						.30	.07	17.55	<.001	1.35
Income						-.15	.09	2.47	.116	.86
Education						-.42	.16	7.04	.008	.66
*Only if Required*										
Control vs. Families	-.07	.48	.02	.892	.94	-.25	.50	.25	.619	.78
Control vs. Government	-.67	.53	1.61	.205	.51	-.79	.54	2.11	.147	.46
Control vs. Choice	-.42	.53	.62	.432	.66	-.52	.55	.90	.343	.60
Political Orientation						.28	.10	8.06	.005	1.32
Income						-.19	.13	2.07	.150	.82
Education						.18	.23	.66	.417	1.20
*Definitely Not*										
Control vs. Families	-1.38	.55	6.41	.011	.25	-1.68	.58	8.44	.004	.19
Control vs. Government	-1.20	.49	6.04	.014	.30	-1.22	.51	5.67	.017	.29
Control vs. Choice	-.59	.45	1.76	.185	.55	-.56	.48	1.37	.243	.57
Political Orientation						.37	.10	14.03	<.001	1.45
Income						-.42	.15	7.93	.005	.66
Education						-.33	.22	2.32	.127	.72
Survey 2										
*Wait and See*										
Control vs. Families	.05	.27	.03	.864	1.05	.05	.27	.04	.843	1.06
Control vs. Government	-.19	.27	.50	.480	.83	-.19	.28	.46	.499	.83
Control vs. Choice	-.17	.29	.35	.555	.84	-.17	.29	.34	.560	.84
Control vs. Liberty	-.03	.26	.01	.921	.98	-.06	.26	.05	.822	.94
Political Orientation						.01	.05	.01	.920	1.01
Income						-.01	.06	.02	.877	.99
Education						-.43	.11	16.57	<.001	.65
*Only if Required*										
Control vs. Families	-.09	.32	.07	.786	.92	-.08	.32	.06	.802	.92
Control vs. Government	-.06	.31	.04	.851	.94	-.07	.32	.05	.830	.93
Control vs. Choice	.18	.32	.32	.569	1.20	.19	.32	.35	.554	1.21
Control vs. Liberty	-.98	.37	6.93	.008	.38	-.98	.38	6.90	.009	.37
Political Orientation						.08	.06	2.03	.155	1.08
Income						-.04	.08	.27	.606	.96
Education						-.27	.13	4.51	.034	.76
*Definitely Not*										
Control vs. Families	.26	.35	.55	.459	1.30	.30	.36	.67	.412	1.35
Control vs. Government	-.36	.39	.86	.353	.70	-.30	.40	.56	.453	.74
Control vs. Choice	.38	.36	1.14	.286	1.46	.43	.37	1.39	.239	1.54
Control vs. Liberty	-.42	.38	1.18	.278	.66	-.39	.39	.98	.321	.68
Political Orientation						.25	.07	12.93	<.001	1.28
Income						-.02	.09	.03	.857	.98
Education						-.77	.14	28.60	<.001	.46

*Note*: The reference category for the vaccine intention variable is “Get it as soon as possible.”

In Survey 2, only the *control* vs. *liberty* coefficient was significant, and only for the comparison between “as soon as possible” and “only if required” responses. From Table 1, we see that compared to the *control* condition, participants in the *liberty* condition were more likely to indicate that they would get the vaccine “as soon as possible” and less likely to say they would get it “only if required.”

### Experimental effects of messages on health-related distrust

The means for *health-related distrust* as a function of condition and survey are presented in Table 1. Message condition effects on distrust were examined via linear regression analyses using the same sets of dummy codes as for the vaccine attitude variables. Results are presented in Table 5. In Survey 1, the *control vs. families* and the *control vs. government* coefficients only approached marginal significance in Model 2, indicating nonsignificant trends toward higher distrust in the *control* condition than in the *families* and *government* conditions. In Survey 2, the *control* vs. *liberty* contrast was significant in both Models, indicating that participants reported higher levels of distrust in the *control* condition than in the *liberty* condition.

**Table 5:** Regression analyses predicting distrust with messaging conditions and demographic covariates.

	Model 1				Model 2			
Variable	*β*	*SE*	*t*	*p*	*β*	*SE*	*t*	*p*
Survey 1								
*Condition Codes*								
Control vs. Families	-.05	.32	-0.77	.443	-.11	.29	-1.95	.052
Control vs. Government	-.07	.32	-1.16	.247	-.10	.29	-1.71	.097
Control vs. Choice	.00	.33	0.01	.995	-.04	.30	-0.56	.523
*Demographic Covariates*								
Political Orientation					.45	.05	9.42	<.001
Income					-.13	.07	-2.68	.012
Education					.04	.12	0.64	.469
Survey 2								
*Condition Codes*								
Control vs. Families	-.03	.19	-0.73	.468	-.03	.17	-0.68	.500
Control vs. Government	-.03	.19	-0.60	.548	-.01	.18	-0.26	.797
Control vs. Choice	-.02	.19	-0.38	.704	-.01	.18	-0.19	.846
Control vs. Liberty	-.13	.19	-2.80	.005	-.11	.18	-2.62	.009
*Demographic Covariates*								
Political Orientation					.33	.03	9.74	<.001
Income					-.11	.04	-3.04	.002
Education					.09	.07	2.35	.019

### Vaccine attitudes and health-related distrust

As trust emerges so centrally in understanding health behavior, we also report the relationships, across all conditions, between our various measures of vaccine attitudes and the measure of health-related distrust. In Survey 1, *health-related distrust* was negatively correlated with *vaccine likelihood*, *r*(369)=−.57, *p* < .001, indicating that lower trust was associated with being less likely to get vaccinated. A One-Way ANOVA predicting distrust with the categorical *vaccine intention* variable was also highly significant, *F*(3, 367)=27.22, *p* < .001. Tukey’s HSD post-hoc tests indicated that *health-related distrust* was significantly lower for participants who said they would get it as soon as possible (*M* = 2.68) than for those who would wait and see (*M* = 3.89), than those who would get it only if required (*M* = 4.25), and than those who indicated they would definitely not get it (*M* = 4.71). All three comparisons were significant at *p* < .001.

In Survey 2, *health-related distrust* was again negatively correlated with *vaccine likelihood*, *r*(793)=−.45, *p* < .001, indicating that lower trust was associated with being less likely to get vaccinated. A One-Way ANOVA predicting distrust with the categorical *vaccine intention* variable was also significant, *F*(3, 791)=16.44, *p* < .001. Tukey’s HSD post-hoc tests indicated that *health-related distrust* was significantly lower for participants who said they would get it as soon as possible (*M* = 3.92) relative to those who would get it only if required (*M* = 4.46), *p* < .001, and relative to those who indicated they would definitely not get it (*M* = 5.18), *p* < .001.

## Discussion

These findings provide support for our hypothesis that public health messaging that affirms peoples’ social experiences and values would be more influential than standard information-based messaging alone. Across outcome variables, the most impactful messages were those that affirmed distrust in the government, the importance of family, and value of personal liberty. Here, we briefly summarize key results and discuss implications for future research in this area.

In Survey 1, relative to the control condition, validating peoples’ wariness about the government increased their expressed likelihood of getting vaccinated, and, ironically, also marginally *increased* health-related trust. Emphasizing the importance of family in peoples’ lives also significantly increased the likelihood of getting vaccinated.

The attitudes of individuals in Survey 2 were somewhat differently influenced by message content. Emphasizing governmental distrust continued to shift individuals’ behavioral intentions in the direction of greater vaccine likelihood, but highlighting the importance of family was not a significant predictor for any of our variables. In contrast, a new condition that emphasized liberty concerns resulted in higher vaccine likelihood, both for oneself and for one’s children, as well as greater health-related trust.

Consistent with prior work on attitude change, the present findings support the notion that health care attitudes and behaviors are much more than the outcome of rational deliberation – they function as profound expressions of individuals’ personal and cultural values [21–23]. While direct communication of health information to the public remains a laudable and essential goal, how individuals or subcultural constituencies interpret this information and how they choose to act or not act on the basis of it will always remain socially-motivated— a function of what is deemed important, moral, and identity-affirming. Because such motivations are so profoundly influential in human culture and psychology, we argue that it is immensely important to promote inclusivity and tolerance of multiple moral and ideological frameworks, and to appeal to the functions of attitudes from multiple perspectives.

Practically speaking, our research findings suggest that framing vaccine information in terms of locally-relevant, or culturally-specific social identities and values is likely to influence vaccination behavior through a variety of mechanisms. In terms of the 5/7C framework for vaccination readiness [45,46], individuals who experience a message as identity-affirming may be: (1) more *confident* or trusting of the institutional source of the message, (2) more *compliant* because the source feels like one of “their people,” and (3) less *constrained* because getting vaccinated feels psychologically consistent with one’s sense of self, rather than a betrayal of one’s social values. Taking our data in conjunction with emerging work in this area [21–23], we argue that attending to such sociocultural factors through outreach and tailored messaging campaigns is foundational for effective public health policy.

### Limitations

One notable limitation of our research was the sampling of relatively low-paid participants obtained via the Amazon MTurk system. While lower pay may not necessarily influence response quality [47], it is considered to be one of a number of increasing concerns with the acceleration of online human-subjects research [48]. Most relevant to the present investigation, a primary consequence of low data quality in experiments conducted on-line is likely to be a weakening of treatment effects [49,50], making it difficult to determine if the null or weak effects observed here are due to ineffective messaging or less engaged participants. Additionally, while MTurk samples are generally demographically diverse and display strong psychometric properties (e.g., test-retest reliability, experimental replication) [32,33], representativeness and data quality have been increasing concerns with the acceleration of online human-subjects research [48]. Additionally, MTurk samples have been shown to be limited in their representativeness of the health behaviors of the general population (e.g., Qureshi, Edelen, Hilton, Rodriguez, Hays, & Herman, 2022; Walters, Christakis, & Wright, 2018 [51,52]). While the vaccination likelihood data reviewed above demonstrated that our samples were fairly representative of the U.S. at the time, demographic comparisons indicated that our samples were younger and more educated than the general U.S. adult population.

## Conclusions

While the message framing effects observed in the present investigation were modest, they could conceivably have substantial effects on vaccination behaviors at scale. We conclude with words of caution against using evidence such as this as a simple tool of persuasion or manipulation. In our work studying COVID-19 responses, our goals have been to listen and categorize people’s important values and perceptions of risk as a means to enrich the multiplicity of public discourse, and to take seriously how and why vaccine hesitancy acts as a reasonable way to express the situatedness of one’s personal and collective social life. Understanding and empathizing with the authentic experiences of a vaccine-hesitant public is likely to make communication around health policy issues less defensive, less volatile, and more open to new information.

## Supporting information

S1 Appendix Vaccine messaging condition descriptions. (DOCX)
