# Supplementary material for: Incorporating public values into public health communications: Effects of value affirmations on intentions to vaccinate and trust in the U.S
Source: PLoS One. 2026 Sep 16;21(9):e0356783. doi: 10.1371/journal.pone.0356783 (PMC13580949; doi:10.1371/journal.pone.0356783)
Supplement: Supplementary file 1 [file pone.0356783.s001.docx]

**Appendix**

Participants were presented with the following information about COVID-19 vaccines. Immediately beforehand, one of the following slogans was presented in very large, bold font:

**You don’t have to trust the government, here are the facts:** (government condition)

**Everybody wants to get back to their families** (families condition)

**It’s your life, it’s your choice** (choice condition)

**COVID and politicians: Don’t tread on me** (liberty condition)
