# Supplementary material for: Incorporating public values into public health communications: Effects of value affirmations on intentions to vaccinate and trust in the U.S
Source: PLoS One. 2026 Sep 16;21(9):e0356783. doi: 10.1371/journal.pone.0356783 (PMC13580949; doi:10.1371/journal.pone.0356783)
Supplement: Supplementary file 2 [file pone.0356783.s003.docx]

April 8, 2026

Thank you for the opportunity to revise our submission, “Incorporating Public Values into Public Health Communications: Effects of Value Affirmations on Intentions to Vaccinate and Trust in the U.S.,” (PONE-D-25-67334) for consideration for publication in *PLOS One.*

Below, we highlight the changes we have made in response to feedback from the editorial staff and reviewers.

1. We have closely adhered to the style guidelines for the journal and have made adjustments in headings, subheadings, citations, and references.
2. The data for this project (*Incorporating Public Values into Public Health*) is now publicly available at the Open Science Framework at <https://osf.io/xhm2w>
3. We have deleted the funding statement from the Acknowledgments section of the manuscript. As such, we no longer require an Acknowledgements section. We are happy with the way the current Funding Statement reads. No edits are needed from our perspective.
4. We have included captions for our Supporting Information files at the end of our manuscript and updated in-text citations to match accordingly.
5. For several different reasons, Reviewer #1 requests that we better integrate our unique contributions into the existing literature on models of vaccine attitudes and behavior. We have done so at several points in the introduction, commenting on how our work goes beyond older, conventional models (Theory of Planned Behavior, Health Belief Model), and contributes to a more nuanced understanding of specific mechanisms in the 5/7C predictors. We maintain the social psychological discussion on attitude change in the introduction, as we think this provides a robust, empirically-grounded explanation for hypothesizing our message framing effects.
6. Reviewer #1 suggests that the abstract should emphasize the magnitudes and effect sizes of the variables employed in the study. We now highlight the small effect sizes of our messaging conditions. Relatedly, this reviewer questions whether we only used confidence intervals to estimate significance. We emphasize effect sizes at several points (including by reporting standardized Betas in the linear regressions) to address this concern. As with many other studies on message framing, we are transparent that the effects observed are modest.
7. Reviewer #1 requests a more detailed discussion of the development and validity of the various measures of trust and vaccine attitudes. We now accomplish this in the Methods section, providing more details on the rationale for the measures and how they have been used in prior research.
8. Reviewer #2 highlights the need for a Statistical analysis section. We have provided this now as a subsection under the Methods. It includes a rationale for the logistic regression model (Reviewer #1), a verification that we used two-tailed tests (Reviewer #1), and the description of the dummy coding and model building procedure for all regressions (Reviewer #2).
9. Reviewer #1 requests some commentary on response biases and the representativeness of the samples. We have addressed this by comparing our demographic and vaccine intention data to nationally representative data from the time of our study. Additionally, we include a more extensive critique of MTurk responses in the Discussion.
10. We now discuss the generalizability of our findings in the Results and Discussion sections (Reviewer #1).
11. We now include additional reflection in the Discussion section on how our work can be practically applied to enhance our understanding and application of existing models of vaccine behavior (Reviewer #1).
12. The Intro, Methods, and Discussion sections all expand the connection between this project and similar research (Reviewer #2).
13. We have expanded our discussion of the limitations of the manuscript, including limits on generalizability and the potential biases associated with MTurk samples.
14. As suggested by Reviewer #2, we have moved the overview of our discourse analysis to the Methods section, and focused the end of the Introduction more explicitly on the aims of the study.
