# Supplementary material for: Incorporating public values into public health communications: Effects of value affirmations on intentions to vaccinate and trust in the U.S
Source: PLoS One. 2026 Sep 16;21(9):e0356783. doi: 10.1371/journal.pone.0356783 (PMC13580949; doi:10.1371/journal.pone.0356783)
Supplement: Peer review history

# Peer review: Decision Letter 0

**DOI:** 10.1371/journal.pone.0356783.r001

**Authors:** Katherine Demi Kokkinias

5 Mar 2026

Dear Dr. Wolsko,

Thank you for submitting your manuscript to PLOS ONE. After careful consideration, we feel that it has merit but does not fully meet PLOS ONE’s publication criteria as it currently stands. Therefore, we invite you to submit a revised version of the manuscript that addresses the points raised during the review process.

==============================

The manuscript has been evaluated by two reviewers, and their comments are available below.

The reviewers have raised a number of major concerns. The reviewers would like the authors to explain various aspects of the methodology in more detail including details on how the survey instrument was validated, logistical regression, and how missing data and response bias were accounted for. The reviewers would also like a more robust evaluation of the limitations of the study and the theoretical framework used to guide this study. The reviewers have also made suggestions to improve the clarity of the manuscript. Please also reflect on the limitations and potential bias on the survey platform, Amazon MTurk.

Could you please carefully revise the manuscript to address all comments raised?

==============================

Please submit your revised manuscript by Apr 17 2026 11:59PM. If you will need more time than this to complete your revisions, please reply to this message or contact the journal office at plosone@plos.org. When you're ready to submit your revision, log on to https://www.editorialmanager.com/pone/ and select the 'Submissions Needing Revision' folder to locate your manuscript file.

- A letter that responds to each point raised by the academic editor and reviewer(s). You should upload this letter as a separate file labeled 'Response to Reviewers'.
- A marked-up copy of your manuscript that highlights changes made to the original version. You should upload this as a separate file labeled 'Revised Manuscript with Track Changes'.
- An unmarked version of your revised paper without tracked changes. You should upload this as a separate file labeled 'Manuscript'.

If you would like to make changes to your financial disclosure, please include your updated statement in your cover letter. Guidelines for resubmitting your figure files are available below the reviewer comments at the end of this letter.

If applicable, we recommend that you deposit your laboratory protocols in protocols.io to enhance the reproducibility of your results. Protocols.io assigns your protocol its own identifier (DOI) so that it can be cited independently in the future. For instructions see: https://journals.plos.org/plosone/s/submission-guidelines#loc-laboratory-protocols. Additionally, PLOS ONE offers an option for publishing peer-reviewed Lab Protocol articles, which describe protocols hosted on protocols.io. Read more information on sharing protocols at https://plos.org/protocols?utm_medium=editorial-email&utm_source=authorletters&utm_campaign=protocols.

We look forward to receiving your revised manuscript.

Kind regards,

Katherine Demi Kokkinias, Ph.D.

Staff Editor

PLOS One

Journal Requirements:

When submitting your revision, we need you to address these additional requirements.

1. Please ensure that your manuscript meets PLOS ONE's style requirements, including those for file naming. The PLOS ONE style templates can be found at

https://journals.plos.org/plosone/s/file?id=wjVg/PLOSOne_formatting_sample_main_body.pdf and

https://journals.plos.org/plosone/s/file?id=ba62/PLOSOne_formatting_sample_title_authors_affiliations.pdf.

2. Please note that your Data Availability Statement is currently missing [the repository name and/or the DOI/accession number of each dataset OR a direct link to access each database]. If your manuscript is accepted for publication, you will be asked to provide these details on a very short timeline. We therefore suggest that you provide this information now, though we will not hold up the peer review process if you are unable.

3. Thank you for stating the following in the Acknowledgments Section of your manuscript:

“This research was generously supported by grants from the Deschutes County Health Department and the Roundhouse Foundation.”

We note that you have provided additional information within the Acknowledgements Section that is not currently declared in your Funding Statement. Please note that funding information should not appear in the Acknowledgments section or other areas of your manuscript. We will only publish funding information present in the Funding Statement section of the online submission form.

Please remove any funding-related text from the manuscript and let us know how you would like to update your Funding Statement. Currently, your Funding Statement reads as follows:

“-Both authors received the awards from:

-Deschutes County Health Department (https://www.deschutes.org/health)

-Roundhouse Foundation https://roundhousefoundation.org)

-no relevant grant numbers in either case

-the sponsors played no role in design, data collection and analysis, or any aspect of the publishing process”

Please include your amended statements within your cover letter; we will change the online submission form on your behalf.

4. Please include captions for your Supporting Information files at the end of your manuscript, and update any in-text citations to match accordingly. Please see our Supporting Information guidelines for more information: http://journals.plos.org/plosone/s/supporting-information.

5. If the reviewer comments include a recommendation to cite specific previously published works, please review and evaluate these publications to determine whether they are relevant and should be cited. There is no requirement to cite these works unless the editor has indicated otherwise.

[Note: HTML markup is below. Please do not edit.]

Reviewers' comments:

Reviewer's Responses to Questions

**Comments to the Author**

1. Is the manuscript technically sound, and do the data support the conclusions?

Reviewer #1: Partly

Reviewer #2: Yes

**********

2. Has the statistical analysis been performed appropriately and rigorously?

Reviewer #1: Yes

Reviewer #2: Yes

**********

3. Have the authors made all data underlying the findings in their manuscript fully available?

The PLOS Data policy

Reviewer #1: No

Reviewer #2: Yes

**********

4. Is the manuscript presented in an intelligible fashion and written in standard English?

Reviewer #1: Yes

Reviewer #2: Yes

**********

Reviewer #1: Thanks for your invitation. I have major observations regarding this submission. Please see below the key points:

There are many decent papers on this subject from the USA that have been published by reputable journals. Therefore, will this study add something new in the body of existing knowledge is a question for authors to come up with a solid framework to main stream their idea?

The abstract should emphasize the magnitudes and effect sizes of the variables employed in the study. Introduction section is too long. Need to revise and the authors should highlight the problem statement with the support of appropriate literatures.

The study lacks a coherent and justified framework for assessing the predictors of COVID-19 vaccine attitude. There are established frameworks for such an inquiry, such as the Theory of Planned Behavior, the Health Belief Model, or the 5C and 7C psychological predictors of vaccine intention, which were missed in this study and this approach raises questions about the comprehensiveness of the analytical approach.

How the author did conceptualized theoretical concept of the study? The systematic review is important to cite when discussing the development of the questionnaire, and more information should be given. For example

https://doi. org/10.1371/journal.pone.0265496.

I have a major concern regarding the validity of the survey instrument and the absence of detailed information on survey distribution methods. Validity measures of the instrument should be added to the Method section. Was the questionnaire pre-tested? How did the author ensure the validity and reliability of the instrument?

In the statistical analysis it is necessary to describe the logistic regression model developed, the rationale why they are included and the model building strategy. Moreover, it should be indicated if the tests were one-side or two-sided.

Was there any attempt to quantify the response bias: no information about the non-responders has been reported? It would be useful to have some kind of indication of comparability with non-respondents. Is there any population-based data available? How did they differ from those in the sample, how representative is the sample and were the findings representative of the USA?

Did the Authors rely exclusively on confidence intervals to state significance or otherwise? If so, there may be a level of bias.

How did authors ensure the generalizability of the study findings? Mention in the discussion section.

Need to write practical implications (research and policy) of this study.

Did the authors encounter any limitations to the study worth noting?

Reviewer #2: RE: Incorporating Public Values into Public Health Communications: Effects of Value

Affirmations on Intentions to Vaccinate and Trust in the U.S. (PONE-D-25-67334)

The idea of this paper is very interesting, and novel. It is important to test the effects of value affirmation on vaccine acceptance and societal trust, and contribute to the existing knowledge on effective vaccine promotion communication. However, this paper needs minor revisions in order to be suitable for publishing.

Introduction

1. The last paragraph (lines 112-123) describing the discourse analysis better fits the Methods section. Instead of this paragraph, explicitly state the Aim of this study

Materials and methods

1. The sub-section "Statistical analysis" is missing

Results

1. Lines 266-283 (two paragraphs under the sub-title Experimental Effects of Messages on Vaccine Attitudes) should be moved to Materials and methods (Statistical analysis).

Discussion

1. I would suggest to expand the discussion of findings in comparison to similar research

**********

what does this mean?). If published, this will include your full peer review and any attached files.

If you choose “no”, your identity will remain anonymous but your review may still be made public.

**Do you want your identity to be public for this peer review?**  For information about this choice, including consent withdrawal, please see our Privacy Policy

Reviewer #1: **Yes:** Debendra Nath Roy, PhD

Reviewer #2: No

**********

[NOTE: If reviewer comments were submitted as an attachment file, they will be attached to this email and accessible via the submission site. Please log into your account, locate the manuscript record, and check for the action link "View Attachments". If this link does not appear, there are no attachment files.]

To ensure your figures meet our technical requirements, please review our figure guidelines: https://journals.plos.org/plosone/s/figures

You may also use PLOS’s free figure tool, NAAS, to help you prepare publication quality figures: https://journals.plos.org/plosone/s/figures#loc-tools-for-figure-preparation.

NAAS will assess whether your figures meet our technical requirements by comparing each figure against our figure specifications.

# Author comment: Author response to Decision Letter 1

**DOI:** 10.1371/journal.pone.0356783.r002

8 Apr 2026

Thank you for the opportunity to revise our submission, “Incorporating Public Values into Public Health Communications: Effects of Value Affirmations on Intentions to Vaccinate and Trust in the U.S.,” (PONE-D-25-67334) for consideration for publication in PLOS One.

Below, we highlight the changes we have made in response to feedback from the editorial staff and reviewers.

1. We have closely adhered to the style guidelines for the journal and have made adjustments in headings, subheadings, citations, and references.

2. The data for this project (Incorporating Public Values into Public Health) is now publicly available at the Open Science Framework at https://osf.io/xhm2w

3. We have deleted the funding statement from the Acknowledgments section of the manuscript. As such, we no longer require an Acknowledgements section. We are happy with the way the current Funding Statement reads. No edits are needed from our perspective.

4. We have included captions for our Supporting Information files at the end of our manuscript and updated in-text citations to match accordingly.

5. For several different reasons, Reviewer #1 requests that we better integrate our unique contributions into the existing literature on models of vaccine attitudes and behavior. We have done so at several points in the introduction, commenting on how our work goes beyond older, conventional models (Theory of Planned Behavior, Health Belief Model), and contributes to a more nuanced understanding of specific mechanisms in the 5/7C predictors. We maintain the social psychological discussion on attitude change in the introduction, as we think this provides a robust, empirically-grounded explanation for hypothesizing our message framing effects.

6. Reviewer #1 suggests that the abstract should emphasize the magnitudes and effect sizes of the variables employed in the study. We now highlight the small effect sizes of our messaging conditions. Relatedly, this reviewer questions whether we only used confidence intervals to estimate significance. We emphasize effect sizes at several points (including by reporting standardized Betas in the linear regressions) to address this concern. As with many other studies on message framing, we are transparent that the effects observed are modest.

7. Reviewer #1 requests a more detailed discussion of the development and validity of the various measures of trust and vaccine attitudes. We now accomplish this in the Methods section, providing more details on the rationale for the measures and how they have been used in prior research.

8. Reviewer #2 highlights the need for a Statistical analysis section. We have provided this now as a subsection under the Methods. It includes a rationale for the logistic regression model (Reviewer #1), a verification that we used two-tailed tests (Reviewer #1), and the description of the dummy coding and model building procedure for all regressions (Reviewer #2).

9. Reviewer #1 requests some commentary on response biases and the representativeness of the samples. We have addressed this by comparing our demographic and vaccine intention data to nationally representative data from the time of our study. Additionally, we include a more extensive critique of MTurk responses in the Discussion.

10. We now discuss the generalizability of our findings in the Results and Discussion sections (Reviewer #1).

11. We now include additional reflection in the Discussion section on how our work can be practically applied to enhance our understanding and application of existing models of vaccine behavior (Reviewer #1).

12. The Intro, Methods, and Discussion sections all expand the connection between this project and similar research (Reviewer #2).

13. We have expanded our discussion of the limitations of the manuscript, including limits on generalizability and the potential biases associated with MTurk samples.

14. As suggested by Reviewer #2, we have moved the overview of our discourse analysis to the Methods section, and focused the end of the Introduction more explicitly on the aims of the study.

Attachment Submitted filename: Response to Reviewers.docx

# Peer review: Decision Letter 1

**DOI:** 10.1371/journal.pone.0356783.r003

**Authors:** Tevfik Murat Yildirim

9 Aug 2026

Incorporating Public Values into Public Health Communications: Effects of Value Affirmations on Intentions to Vaccinate and Trust in the U.S.

PONE-D-25-67334R1

Dear Dr. Wolsko,

We’re pleased to inform you that your manuscript has been judged scientifically suitable for publication and will be formally accepted for publication once it meets all outstanding technical requirements.

Within one week, you’ll receive an e-mail detailing the required amendments. When these have been addressed, you’ll receive a formal acceptance letter and your manuscript will be scheduled for publication.

An invoice will be generated when your article is formally accepted. Please note, if your institution has a publishing partnership with PLOS and your article meets the relevant criteria, all or part of your publication costs will be covered. Please make sure your user information is up-to-date by logging into Editorial Manager at Editorial Manager® and clicking the ‘Update My Information' link at the top of the page. For questions related to billing, please contact billing support.

If your institution or institutions have a press office, please notify them about your upcoming paper to help maximize its impact. If they’ll be preparing press materials, please inform our press team as soon as possible -- no later than 48 hours after receiving the formal acceptance. Your manuscript will remain under strict press embargo until 2 pm Eastern Time on the date of publication. For more information, please contact onepress@plos.org.

Kind regards,

Tevfik Murat Yildirim, PhD

Academic Editor

PLOS One

Additional Editor Comments (optional):

Reviewers' comments:

Reviewer's Responses to Questions

**Comments to the Author**

Reviewer #1: All comments have been addressed

Reviewer #2: All comments have been addressed

**********

2. Is the manuscript technically sound, and do the data support the conclusions?

Reviewer #1: Yes

Reviewer #2: Yes

**********

3. Has the statistical analysis been performed appropriately and rigorously?

Reviewer #1: Yes

Reviewer #2: Yes

**********

4. Have the authors made all data underlying the findings in their manuscript fully available?

The PLOS Data policy

Reviewer #1: Yes

Reviewer #2: Yes

**********

5. Is the manuscript presented in an intelligible fashion and written in standard English?

Reviewer #1: Yes

Reviewer #2: Yes

**********

Reviewer #1: This study focuses on a contemporary and interesting issue and Author addressed all previous comments. No further comments. Best wishes.

Reviewer #2: No comments. No comments. No comments. No comments. No comments. No comments. No comments. No comments.

**********

what does this mean?). If published, this will include your full peer review and any attached files.

If you choose “no”, your identity will remain anonymous but your review may still be made public.

**Do you want your identity to be public for this peer review?**  For information about this choice, including consent withdrawal, please see our Privacy Policy

Reviewer #1: **Yes:** Debendra Nath Roy, Ph.D.

Reviewer #2: No

**********

# Editor report: Acceptance letter

**DOI:** 10.1371/journal.pone.0356783.r004

**Authors:** Tevfik Murat Yildirim

PONE-D-25-67334R1

PLOS One

Dear Dr. Wolsko,

I'm pleased to inform you that your manuscript has been deemed suitable for publication in PLOS One. Congratulations! Your manuscript is now being handed over to our production team.

At this stage, our production department will prepare your paper for publication. This includes ensuring the following:

* All references, tables, and figures are properly cited

* All relevant supporting information is included in the manuscript submission,

* There are no issues that prevent the paper from being properly typeset

You will receive further instructions from the production team, including instructions on how to review your proof when it is ready. Please keep in mind that we are working through a large volume of accepted articles, so please give us a few days to review your paper and let you know the next and final steps.

Lastly, if your institution or institutions have a press office, please let them know about your upcoming paper now to help maximize its impact. If they'll be preparing press materials, please inform our press team within the next 48 hours. Your manuscript will remain under strict press embargo until 2 pm Eastern Time on the date of publication. For more information, please contact onepress@plos.org.

You will receive an invoice from PLOS for your publication fee after your manuscript has reached the completed accept phase. If you receive an email requesting payment before acceptance or for any other service, this may be a phishing scheme. Learn how to identify phishing emails and protect your accounts at https://explore.plos.org/phishing.

If we can help with anything else, please email us at customercare@plos.org.

Thank you for submitting your work to PLOS One and supporting open access.

Kind regards,

PLOS ONE Editorial Office Staff

on behalf of

Dr. Tevfik Murat Yildirim

Academic Editor

PLOS One
